# Utility of ultrasonography for predicting indications for tolvaptan in patients with autosomal dominant polycystic kidney disease

**DOI:** 10.1007/s10396-022-01261-z

**Published:** 2022-11-05

**Authors:** Hiroko Iijima, Toshifumi Tada, Mariko Hashimoto, Takashi Nishimura, Masato Kiriki, Akiko Higashiura, Aya Iwasaki, Michino Honda, Yasuyuki Nagasawa, Koichiro Yamakado

**Affiliations:** 1 Division of Gastroenterology and Hepatology, Department of Internal Medicine, Hyogo Medical University, 1-1 Mukogawa-cho, Nishinomiya, Hyogo 663-8501 Japan; 2 Ultrasound Imaging Center, Hyogo Medical University, Nishinomiya, Hyogo Japan; 3 Department of Internal Medicine, Japanese Red Cross Society Himeji Hospital, Himeji, Hyogo Japan; 4 Department of Radiologic Technology, Hyogo Medical University, Nishinomiya, Hyogo Japan; 5 Department of General Internal Medicine, Hyogo Medical University, Nishinomiya, Hyogo Japan; 6 Department of Radiology, Hyogo Medical University, Nishinomiya, Hyogo Japan

**Keywords:** Autosomal dominant polycystic kidney disease, Ultrasonography, Total kidney volume, Tolvaptan

## Abstract

**Purpose:**

Tolvaptan is the first approved treatment for autosomal dominant polycystic kidney disease (ADPKD) that targets a mechanism directly contributing to the development and growth of renal cysts. We investigated the ability of ultrasonography to predict total kidney volume (TKV) of 750 mL or more, which is an indication for tolvaptan therapy in patients with ADPKD.

**Methods:**

A total of 46 patients with ADPKD were evaluated. The most statistically appropriate measurement based on ultrasonography for predicting TKV determined by computed tomography (CT) was assessed.

**Results:**

TKV determined by CT was 796.8 (508.8–1,560.3) mL. The median length, anteroposterior distance, and mediolateral distance determined using ultrasonography were 15.7 cm, 7.6 cm, and 7.6 cm in the left kidney, and 13.4 cm, 6.9 cm, and 7.2 cm in the right kidney, respectively. Multivariate regression analysis showed that total kidney length (left and right) [variance inflation factor (VIF), 9.349] and total mediolateral distance (left and right) (VIF, 3.988) were independently associated with TKV. The correlation (*r*) between the logarithm of TKV determined by CT and total mediolateral distance determined using ultrasonography was 0.915 (*p* < 0.001). The linear regression equation was log (total kidney volume) = 1.833 + 0.075 × total mediolateral distance (left and right) based on ultrasonography. The area under the receiver operating characteristic curve for total mediolateral distance determined using ultrasonography to predict TKV of 750 mL or more was 0.989. Using the total mediolateral distance cut-off value of 14.2 cm, the sensitivity and specificity were 96.0% and 100.0%, respectively.

**Conclusion:**

Total mediolateral distance determined using ultrasonography can predict TKV in patients with ADPKD.

## Introduction

Autosomal dominant polycystic kidney disease (ADPKD) is the most common inherited renal disease [1, 2]. Although ADPKD is a progressive disease, there is inter-individual variability in the rate of progression [3]. Thus, a readily available imaging method to determine the rate of progression in each patient is valuable for clinical management of ADPKD [4].

Quantifying renal enlargement might lead to a useful marker of ADPKD progression [5, 6]. This concept has been applied to imaging-based classifications [5] used to individualize prognosis and make treatment decisions [7–9]. Current recommendations call for all patients with ADPKD to receive an evaluation of renal size and volume as part of their initial assessment [7–10].

Tolvaptan, a selective arginine vasopressin receptor type 2 antagonist, is the first approved treatment for ADPKD that targets a mechanism directly contributing to the development and growth of renal cysts [11, 12]. In Japan, tolvaptan was approved for use in the treatment of “progressive ADPKD [total kidney volume (TKV) ≥ 750 mL or annual growth rate of TKV ≥ 5%]” in March 2014. Therefore, it is important to detect TKV ≥ 750 mL using a diagnostic imaging method that is as noninvasive and accurate as possible.

For assessment of TKV, computed tomography (CT) is an accurate diagnostic tool in patients with ADPKD [13]. It has been proven to be of similar diagnostic value as magnetic resonance imaging (MRI) in TKV measurement [14, 15]. However, CT requires patient exposure to ionizing radiation and nephrotoxic iodinated contrast agents. In addition, nephrogenic systemic fibrosis can affect patients with pre-existing impaired renal function after gadolinium administration for MRI [15]. Therefore, it is necessary to identify more noninvasive imaging methods for predicting TKV in patients with ADPKD.

In this study, we investigated whether ultrasonography can be used for TKV assessment in patients with ADPKD. In addition, we determined the most appropriate ultrasound kidney measurement and its cut-off value for predicting when tolvaptan administration is indicated in patients with ADPKD.

## Materials and methods

### Patients

A total of 136 patients with ADPKD underwent ultrasonography at the Hyogo Medical University between June 2013 and March 2020. Of these, 46 met the following eligibility criteria and were enrolled in this study: (1) abdominal CT was performed, (2) TKV based on CT was available, and (3) the duration between ultrasonography and CT examinations was within 1.5 years. Patient age, sex, height, and weight were recorded at baseline. Fasting blood counts and biochemistry tests were conducted using standard methods.

ADPKD was diagnosed using previously established diagnostic criteria [17]. The patients consisted of 21 (45.7%) females and 25 (54.3%) males with a median age of 66.5 (52.8–76.8) years. The median estimated glomerular filtration rate (eGFR) was 49.0 (8.0–70.8) mL/min/1.73 m^2^ (Table 1).

**Table 1:** Characteristics of study patients (*n* = 46)

Age (years)*	66.5 (52.8–76.8)
Sex (female/male)	21/25
BMI (kg/m^2^)*	21.9 (19.4–25.0)
eGFR (mL/min/1.73 m^2^)*	49.0 (8.0–70.8)

*BMI* body mass index, *eGFR* estimated glomerular filtration rate

*Values are expressed as medians (interquartile range)

The study protocol complied with the Helsinki Declaration. It was approved by the institutional review board of the Hyogo Medical University (number 3516). We used the opt-out method to obtain informed consent in this study.

### Measurement of kidney size and TKV by CT

**Fig. 1:**
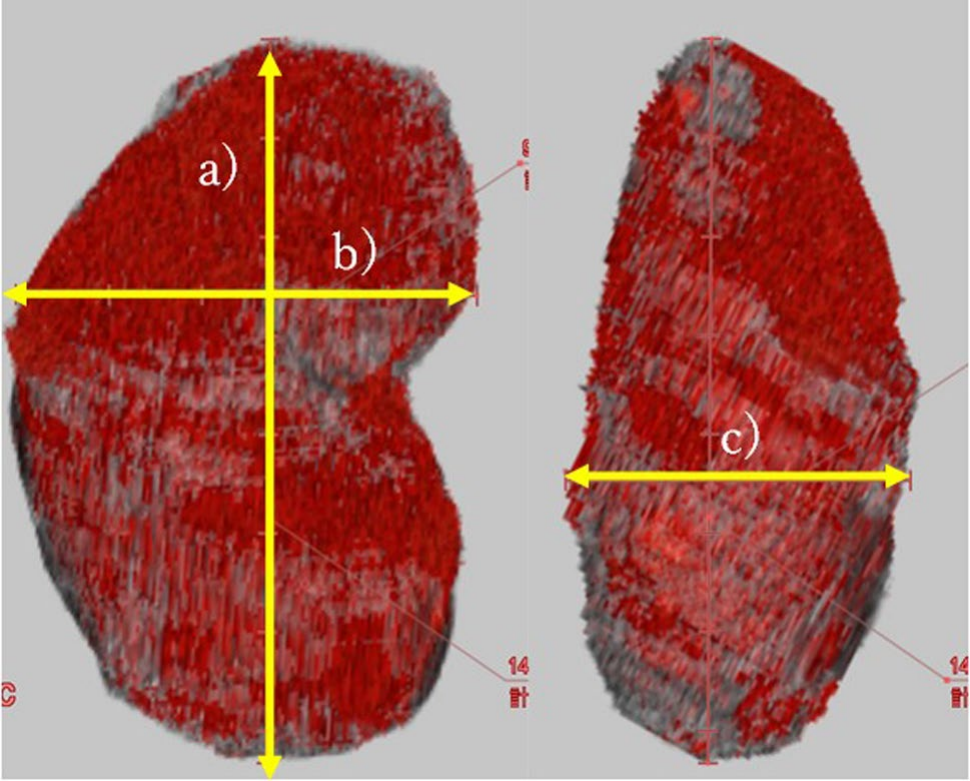
Measurement of kidney size and volume using Workstation (Ziostation 2) in a male patient in his 20 s. **a**. Major axis: 147 mm. **b**. Minor axis: 99 mm. **c**. Width: 74 mm. TKV was 504 mL

CT (SOMATOM Definition Edge; Siemens Healthcare, Forchheim, Germany) and Workstation (Ziostation 2; Ziosoft, Tokyo, Japan) were used to measure kidney size and TKV. Axial CT images with a 5-mm slice thickness were traced from the upper pole to the lower pole (Fig. 1). A single experienced radiology technician (K.M., with over 25 years of experience in CT imaging) who was blinded to the patients’ clinical and ultrasonography data determined the major axis, minor axis, and width (Fig. 1a–c).

### Measurement of kidney size by ultrasonography

**Fig. 2:**
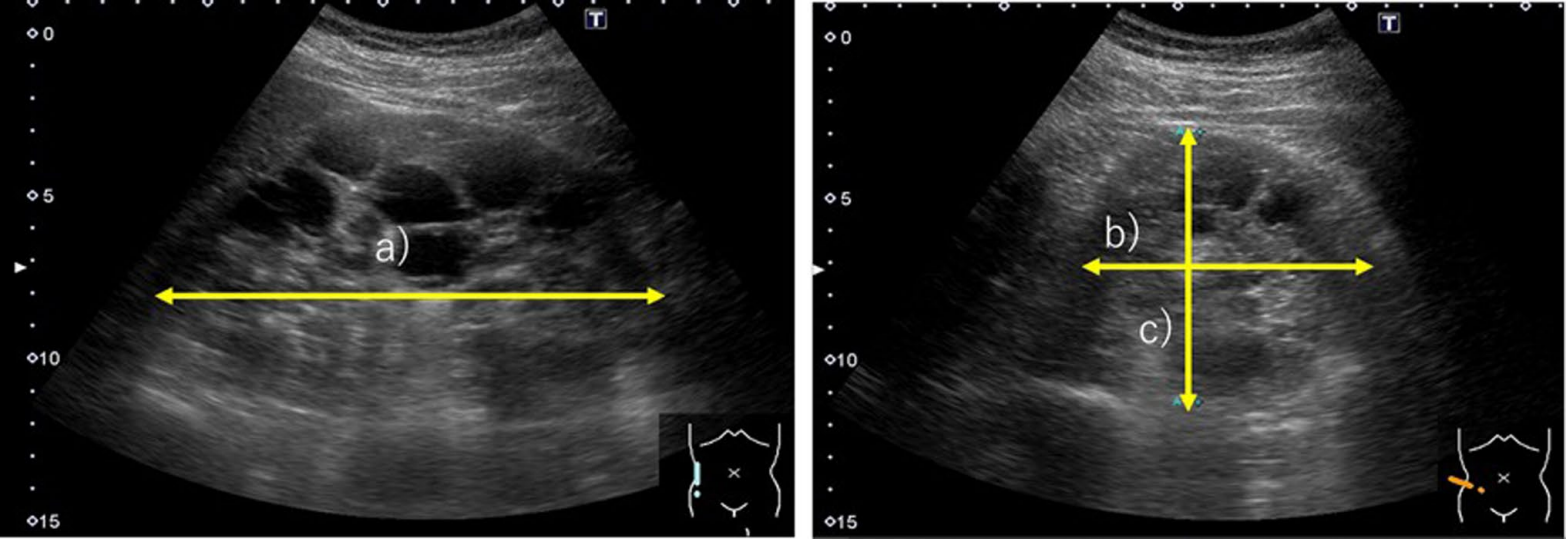
Measurement of kidney size using ultrasonography in a male patient in his 20 s. **a**. Maximum renal height (length): 151 mm. **b**. Maximum anteroposterior distance: 82 mm. **c**. Maximum mediolateral distance: 64 mm. TKV was 415 mL

B-mode and color Doppler ultrasound examinations were performed in the supine, lateral, and posterolateral positions to provide optimal images. Patients were asked to fast and empty their bladders before the examination. Measurements were made during a breath hold using an Aplio i800 (Canon Medical Systems, Tokyo, Japan) with a convex probe (PVI-475BX; Canon Medical Systems, Tokyo, Japan). A single experienced sonologist (M.H., with over 20 years of experience in renal ultrasonography) who was blinded to the patients’ clinical and CT data determined the maximum renal height (length) in the sagittal plane (Fig. 2a) and measured the maximum anteroposterior distance in the same plane (Fig. 2b), perpendicular to the plane used to measure the maximum renal height. The maximum mediolateral distance was determined in the axial plane (Fig. 2c) [16]. To measure the maximum mediolateral distance, the renal hilum was identified with color Doppler ultrasound. Measurements were made in the plane in which the renal vasculature in the hilum was as parallel as possible to the axial plane.

### Statistical analysis

Continuous variables are expressed as medians (interquartile range). The Mann–Whitney *U* test or Kruskal–Wallis test was used for continuous variables.

Single and multiple regression analyses (univariate and multivariate analyses) were performed with TKV as the objective variable, and age, body mass index (BMI), and total size of the left and right kidneys as covariates. If the objective variable was not normally distributed according to the Kolmogorov–Smirnov test, it was logarithmically transformed. The results of the regression models are reported as partial regression coefficients and 95% confidence intervals. The adjusted coefficient of determination was used to evaluate model performance for multivariate analyses, with adjusted *R*^2^ closer to 1 indicating a better model. The variance inflation factor (VIF) was used to assess for multicollinearity in multivariate regression models. VIF > 10 was defined as serious multicollinearity. VIF > 4 was considered a cause for concern [17].

Correlations between TKV based on CT and kidney size determined by ultrasonography were analyzed using the Pearson product–moment correlation coefficient (*r*), which was classified as minimal (|*r*|< 0.2), weak (|*r*|= 0.2–0.4), moderate (|*r*|= 0.4–0.7), or strong (|*r*|≥ 0.7) [18].

The diagnostic value of the statistically selected kidney size based on multivariate analysis was evaluated using receiver operating characteristic (ROC) curve analysis. In this study, we defined the TKV cut-off to be 750 mL, which is an indication for tolvaptan therapy in ADPKD. We calculated the sensitivity, specificity, accuracy, positive predictive value, and negative predictive value using the maximum Youden index (sensitivity + specificity − 1) of the cut-off level [19] when comparing TKV and kidney size. Diagnostic values were classified as low [area under the ROC curve (AUROC) = 0.50–0.70], moderate (AUROC = 0.70–0.90), or high (AUROC = 0.90–1.0) [20].

Statistical significance was defined as *p* < 0.05. Statistical analyses were performed with EZR version 1.53 (Saitama Medical Center, Jichi Medical University, Saitama, Japan), which is a graphical user interface for R (The R Foundation for Statistical Computing, Vienna, Austria) [21]. More precisely, it is a modified version of the R commander designed to add statistical functions frequently used in biostatistics.

## Results

### Kidney size and volume determined by CT

Kidney size and volume measured using CT are shown in Table 2. The volume of the left kidney was 431.8 (255.1–791.5) mL. The volume of the right kidney was 374.2 (230.1–754.5) mL. TKV was 796.8 (508.8–1,560.3) mL. There were 25 (54.3%) patients with TKV ≥ 750 mL.

**Table 2:** Kidney size determined by CT and ultrasonography, and volume determined by CT

	Right			Left		
	CT	Ultrasonography	*p* value	CT	Ultrasonography	*p* value
Length (cm)	13.9 (11.7–16.7)	13.4 (11.4–18.1)	0.963	14.6 (11.8–17.3)	15.7 (12.4–20.3)	0.246
Anteroposterior distance (cm)	8.3 (6.3–10.6)	7.2 (6.0–10.9)	0.436	7.2 (6.1–9.5)	7.6 (6.1–10.1)	0.515
Mediolateral distance (cm)	7.5 (6.1–10.2)	6.9 (5.7–8.7)	0.915	8.0 (6.3–9.7)	7.6 (5.9–8.5)	0.264
Volume (mL)	374.2 (230.1–754.5)			431.8 (255.1–791.5)		

*CT* computed tomography

### Kidney size measured by ultrasonography

Kidney size measured using ultrasonography is shown in Table 2. The median length, anteroposterior distance, and mediolateral distance of the left kidney were 15.7 cm, 7.6 cm, and 7.6 cm, respectively. The median length, anteroposterior distance, and mediolateral distance of the right kidney were 13.4 cm, 6.9 cm, and 7.2 cm, respectively.

### Univariate and multivariate analyses of TKV

The *p* value of the Kolmogorov–Smirnov test for TKV was 0.031. Therefore, TKV values were logarithmically transformed. Table 3 shows the results of the univariate and multivariate regression analyses. In the multivariate analysis, the adjusted *R*^2^ of the multivariate model was 0.932. Total kidney length (left and right) and total mediolateral distance (left and right) were independently associated with TKV. VIF values for age, BMI, total length, total anteroposterior distance, and total mediolateral distance were 1.084, 1.045, 9.349, 10.046, and 3.988, respectively, which suggested there was cause for concern with respect to multicollinearity. Therefore, we considered total mediolateral distance (left and right) measured by ultrasonography as a reasonable parameter that was correlated with TKV determined by CT.

**Table 3:** Univariate and multivariate analyses^a^

	Single regression analysis			Multiple regression analysis			
	Partial regression coefficient	95% Confidence interval	*p* value	Partial regression coefficient	95% Confidence interval	*p* value	VIF
Intercept				1.616	1.318–1.914	< 0.001	
Age	−0.002	−0.009 to 0.005	0.551	0.000	−0.001 to 0.002	0.880	1.084
BMI	0.015	−0.017 to 0.046	0.351	0.002	−0.006 to 0.010	0.635	1.045
Length (cm)	0.035	0.030–0.039	< 0.001	0.026	0.015–0.038	< 0.001	9.349
Anteroposterior distance (cm)	0.045	0.039–0.052	< 0.001	−0.002	−0.015 to 0.012	0.800	10.046
Mediolateral distance (cm)	0.075	0.065–0.084	< 0.001	0.034	0.020–0.048	< 0.001	3.988

*BMI* body mass index, *VIF* variance inflation factor, *TKV* total kidney volume

^a^TKV was used as the objective variable in the single and multiple regression analyses

### Correlation between TKV determined by CT and total mediolateral distance (left and right) measured by ultrasonography

**Fig. 3:**
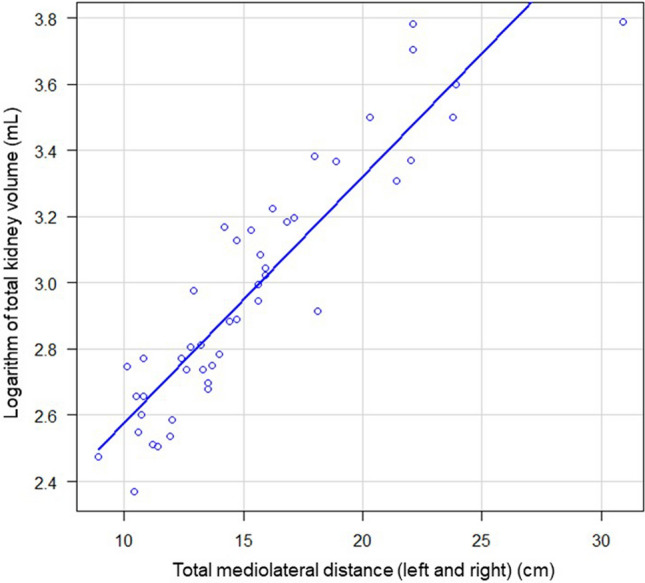
Correlation between TKV determined by CT and total mediolateral distance (left and right) measured by ultrasonography. The correlation (*r*) between the logarithm of TKV determined by CT and total mediolateral distance measured by ultrasonography was 0.915 (95% confidence interval 0.851–0.952) (*p* < 0.001)

The correlation (*r*) between the logarithm of TKV determined by CT and total mediolateral distance measured by ultrasonography was 0.915 (*p* < 0.001), corresponding to a strong correlation (Fig. 3). The linear regression equation was log (TKV) = 1.833 + 0.075 × total mediolateral distance (left and right) measured by ultrasonography.

### Performance of total anteroposterior distance (left and right) measured by ultrasonography for diagnosing TKV of 750 mL or more

**Fig. 4:**
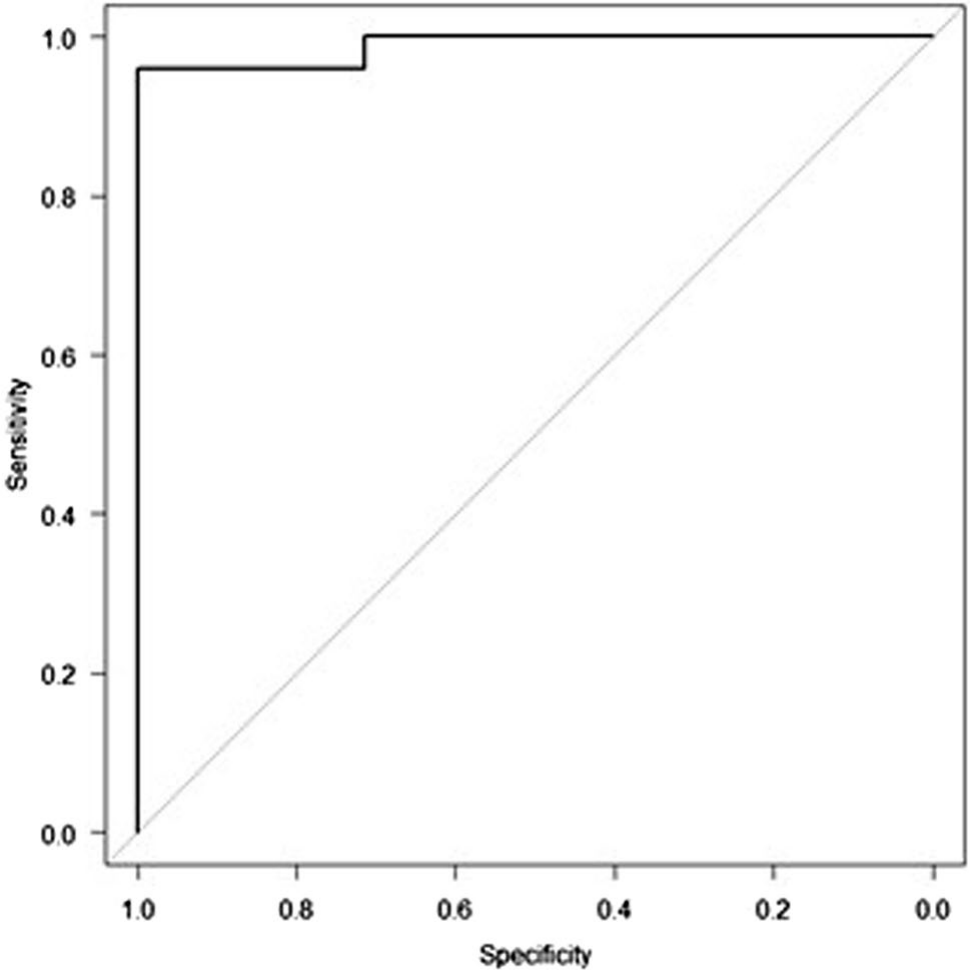
ROC curve of total mediolateral distance measured by ultrasonography for diagnosing TKV of 750 mL or more. The AUROC for total mediolateral distance measured by ultrasonography was 0.989 (95% confidence interval 0.965–1.000)

The ROC curve for total mediolateral distance measured by ultrasonography to diagnose TKV of 750 mL or more is shown in Fig. 4. The AUROC of total mediolateral distance measured by ultrasonography was 0.989.

The cut-off value for total mediolateral distance measured by ultrasonography according to the Youden index was 14.2 cm. The sensitivity, specificity, accuracy, positive predictive value, and negative predictive value of total mediolateral distance measured by ultrasonography for diagnosing TKV of 750 mL or more using this cut-off value are shown in Table 4.

**Table 4:** Diagnosing total kidney volume of 750 mL based on total mediolateral distance measured by ultrasonography with cut-off value of 14.2 cm

Sensitivity	96.0% (95% confidence interval 76.9–99.9)
Specificity	100.0% (95% confidence interval 77.2–100.0)
PPV	100.0% (95% confidence interval 79.6–100.0)
NVP	95.6% (95% confidence interval 77.2–99.9)
Accuracy	97.8% (95% confidence interval 88.5–99.9)

*PPV* positive predictive value, *NPV* negative predictive value

## Discussion

In this study, we demonstrated that specific ultrasound kidney measurements can predict TKV as determined by CT in patients with ADPKD. Multivariate regression analysis, which included total length, total anteroposterior distance, and total mediolateral distance measured using ultrasonography as covariates, showed that total mediolateral distance was significantly and reasonably associated with TKV as determined by CT. The correlation (*r*) between TKV determined by CT and total mediolateral distance measured by ultrasonography was 0.7 or greater, corresponding to a strong correlation. In addition, ROC curve analysis for diagnosing TKV of 750 mL or more, which is an indication for tolvaptan therapy in ADPKD, showed that the AUROC for total mediolateral distance measured by ultrasonography was greater than 0.9, corresponding to high diagnostic value. Furthermore, with 14.2 cm as the cut-off value for total mediolateral distance measured by ultrasonography, the sensitivity and specificity for diagnosing TKV of 750 mL or more were both greater than 95%. Therefore, ultrasonography, a more noninvasive imaging method than CT or MRI, has excellent diagnostic ability for TKV in patients with ADPKD.

Tolvaptan, an oral selective vasopressin V2 receptor antagonist, lowers cAMP levels within the epithelial cells of collecting ducts and distal nephrons, the major sites of cyst development in ADPKD [22]. In the pivotal Tolvaptan Efficacy and safety in Management of Polycystic kidney disease and its Outcomes (TEMPO 3:4) clinical trial, tolvaptan significantly slowed TKV growth by 49.2% relative to placebo (2.8% versus 5.5% per year) [23] in patients with ADPKD, TKV of 750 mL or more, and estimated creatinine clearance of 60 mL per minute or more. Other benefits of tolvaptan treatment included a 26% lower rate of eGFR decline (0.98 mL/min/1.737 m^2^ per year) and adjudicated renal function decline events, equivalent to a 30% reduction in eGFR. Therefore, tolvaptan is recommended for patients with ADPKD who have good renal function and TKV of 750 ml or more. Thus, we investigated ultrasound measurements of the kidney and cut-off values for predicting TKV of 750 mL or more in patients with ADPKD.

Although CT and MRI can accurately diagnose TKV, both imaging modalities have the disadvantage of requiring contrast agent use in patients with chronic kidney disease. Therefore, to predict TKV in patients with ADPKD, it is necessary to establish a more noninvasive, accurate, and repeatable diagnostic imaging method without contrast agent use.

O’Neill et al. [24] investigated the utility of ultrasonography for diagnosing ADPKD as compared with MRI. They found kidney length to be the most reproducible measurement; the correlation with volume determined by MRI was 0.84. In addition, they found that all patients with kidney volume that was less than 700 cm^3^ as measured by ultrasonography had kidney volume that was less than 1,000 cm^3^ as determined by MRI. All patients with kidney volume greater than 1,700 cm^3^ as measured by ultrasonography had kidney volume greater than 1,000 cm^3^ as determined by MRI. Therefore, they concluded that sonographic measurement of kidney volume in patients with ADPKD was inaccurate. In the present study, we found that total mediolateral distance was associated with TKV determined by CT. Although our study included fewer patients than the study by O’Neill et al., one strength of this study is that the most appropriate ultrasound measurement (i.e., total mediolateral distance) was determined using multivariate regression analysis.

The main limitations of this study include its retrospective nature and the relatively small number of patients. Further prospective studies with more patients are warranted. In addition, validation studies were not performed. Further prospective studies that include validation studies are warranted.

## Conclusion

Total mediolateral distance measured by ultrasonography is strongly correlated with TKV assessed by CT in patients with ADPKD. In addition, total mediolateral distance measured by ultrasonography had diagnostic value for predicting TKV of 750 mL or more. Further studies should be conducted to confirm these findings in other populations.
